# IFPTML Mapping of Drug Graphs with Protein and Chromosome Structural Networks vs. Pre-Clinical Assay Information for Discovery of Antimalarial Compounds

**DOI:** 10.3390/ijms222313066

**Published:** 2021-12-02

**Authors:** Viviana Quevedo-Tumailli, Bernabe Ortega-Tenezaca, Humberto González-Díaz

**Affiliations:** 1 Grupo RNASA-IMEDIR, Department of Computer Science, University of A Coruña, 15071 A Coruña, Spain; viviana.quevedo@udc.es (V.Q.-T.); bernabe.ortega@udc.es (B.O.-T.); 2 Research Department, Puyo Campus, Universidad Estatal Amazónica, Puyo 160150, Ecuador; 3 Information and Communications Technology Management Department, Puyo Campus, Universidad Estatal Amazónica, Puyo 160150, Ecuador; 4 Department of Organic and Inorganic Chemistry, University of the Basque Country UPV/EHU, 48940 Leioa, Spain; 5 BIOFISIKA, Basque Centre for Biophysics, CSIC-UPV/EHU, 48940 Leioa, Spain; 6 IKERBASQUE, Basque Foundation for Science, 48011 Bilbao, Spain

**Keywords:** Antimalarial compounds, *Plasmodium* proteome, NCBI-GDV, UniProt, ChEMBL, machine learning, perturbation theory, complex networks

## Abstract

The parasite species of genus *Plasmodium* causes Malaria, which remains a major global health problem due to parasite resistance to available Antimalarial drugs and increasing treatment costs. Consequently, computational prediction of new Antimalarial compounds with novel targets in the proteome of *Plasmodium* sp. is a very important goal for the pharmaceutical industry. We can expect that the success of the pre-clinical assay depends on the conditions of assay per se, the chemical structure of the drug, the structure of the target protein to be targeted, as well as on factors governing the expression of this protein in the proteome such as genes (Deoxyribonucleic acid, DNA) sequence and/or chromosomes structure. However, there are no reports of computational models that consider all these factors simultaneously. Some of the difficulties for this kind of analysis are the dispersion of data in different datasets, the high heterogeneity of data, etc. In this work, we analyzed three databases ChEMBL (Chemical database of the European Molecular Biology Laboratory), UniProt (Universal Protein Resource), and NCBI-GDV (National Center for Biotechnology Information—Genome Data Viewer) to achieve this goal. The ChEMBL dataset contains outcomes for 17,758 unique assays of potential Antimalarial compounds including numeric descriptors (variables) for the structure of compounds as well as a huge amount of information about the conditions of assays. The NCBI-GDV and UniProt datasets include the sequence of genes, proteins, and their functions. In addition, we also created two partitions (c_assayj_ = c_aj_ and c_dataj_ = cd_j_) of categorical variables from theChEMBL dataset. These partitions contain variables that encode information about experimental conditions of preclinical assays (c_aj_) or about the nature and quality of data (c_dj_). These categorical variables include information about 22 parameters of biological activity (c_a0_), 28 target proteins (c_a1_), and 9 organisms of assay (c_a2_), etc. We also created another partition of (c_protj_ = c_pj_) including categorical variables with biological information about the target proteins, genes, and chromosomes. These variables cover32 genes (c_p0_), 10 chromosomes (c_p1_), gene orientation (c_p2_), and 31 protein functions (c_p3_). We used a Perturbation-Theory Machine Learning Information Fusion (IFPTML) algorithm to map all this information (from three databases) into and train a predictive model. Shannon’s entropy measure Sh_k_ (numerical variables) was used to quantify the information about the structure of drugs, protein sequences, gene sequences, and chromosomes in the same information scale. Perturbation Theory Operators (PTOs) with the form of Moving Average (MA) operators have been used to quantify perturbations (deviations) in the structural variables with respect to their expected values for different subsets (partitions) of categorical variables. We obtained three IFPTML models using General Discriminant Analysis (GDA), Classification Tree with Univariate Splits (CTUS), and Classification Tree with Linear Combinations (CTLC). The IFPTML-CTLC presented the better performance with Sensitivity Sn(%) = 83.6/85.1, and Specificity Sp(%) = 89.8/89.7 for training/validation sets, respectively. This model could become a useful tool for the optimization of preclinical assays of new Antimalarial compounds vs. different proteins in the proteome of *Plasmodium*.

## 1. Introduction

Malaria is a major global health concern with cases reported in different regions. At present, the risk areas for contracting this disease are Africa, Central and South America, as well as in some parts of the Caribbean, Asia, Eastern Europe, and the South Pacific. The World Health Organization (WHO) estimated 219 million worldwide reported cases of malaria in 2017. It is an infection of the red blood cells by parasites of the genus *Plasmodium* with the most severe and common forms caused by *Plasmodium falciparum* (*P. falciparum* or *Pf*) and related species such as *Plasmodium vivax* (*P. vivax* or *Pv*), *Plasmodium malariae* (*P. malariae* or *Pm*), and *Plasmodium ovale* (*P. ovale* or *Po*). The most frequent and deadly form is the *Pf*. According to WHO, malaria during pregnancy may cause severecomplications. Emerging parasite resistance to available Antimalarial drugs poses great challenges to treatment.Moreover, the costs have significantly increased in the last few years for the determination and development of the new drug. Tufts Center for the Study of Drug Development estimates an out-of-pocket cost per approved drug in $1861 million for Antimalarial drugs [1,2,3,4].

TheChEMBL database lists >17,750 preclinical assays of Antimalarial compounds. The ChEMBL database about Antimalarial compounds cover multiple biological activity parameters (Inhibition, IC_50_, Activity, etc.), different unique assays only for the protein target of*Pf*organism and is applied to different genes about proteome. In addition, the ChEMBL database compiles datasets of very heterogeneous preclinical assays. We can enrich ChEMBL data with NCBI-GDV and UniProt databases data to obtain information about drug target proteins, chromosomes, and genes. For instance, UniProt includes information related to sequence of proteins.Lastly, NCBI-GDV includes information related to the sequence of genes and the structure of chromosome (DNA sequence, gene adjacency, orientation, etc.) This information may be also relevant for the synthesis of proteins with different functions in the *Pf* [5,6,7,8,9,10,11].

On the other hand, IFPTML models have been used in medicinal chemistry, proteomics, nanotechnology, etc.,for modeling large datasets with Big Data features. IFPTML models combine Information Fusion (IF) techniques with Perturbation Theory (PT) ideas and Machine Learning (ML) algorithms (PT + ML = PTML models). IFPTML modeling is also useful to carry out information fusion of data from diversesources. For instance, we can include data about the protein sequence from GenBank, Metabolic networks, Nanoparticles, or even information about epidemiology data in USA counties, etc. [12,13,14].

In order to develop IFPTML models, we need to use as input variable parameters able to quantify the information about the structural and experimental conditions of assay of all the systems involved (drugs, proteins, gene networks, etc.). In this sense, Shannon’s Entropy information measures introduced by Claude E. Shannon could be extremelyuseful [15]. In fact, Graham, Marrero-Ponce, Barigye, and other researchers, have used different classes of Shannon information values to measure chemical and/or biologically relevant information quantitatively [16,17,18,19,20,21,22,23,24,25,26,27]. González-Díaz and Munteanu combined the idea of Shannon entropy with Markov chains to calculate the Sh(syst)_k_ values, stochastic Shannon’s Entropies of order k^th^, anddifferent molecular systems [28].

**Figure 1:**
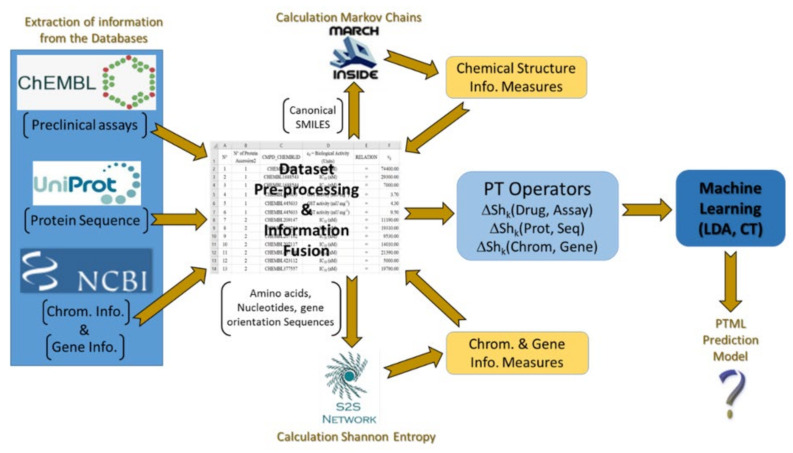
General Workflow of the steps given in this work.

In previous work, we analyzed the proteome/genome and chromosomes of *Pf*using data from NCBI-GDV and UniProt databases [29]. However, this previous work has not considered the possibility of mapping this data vs. preclinical assays of compounds towards the design of new Antimalarials. In addition, there are no reports IFPTML models for Antimalarial compounds considering information from NCBI-GDV, UniProt, and ChEMBL databases at the same time. In this work, we develop a general-purpose IFPTML model for the prediction of new Antimalarial compounds by fusing information from the three different databases. Figure 1 illustrates all the different steps that are included in the general workflow used to obtain this IFPTML model. Firstly, we downloaded all relevant information from the ChEMBL, NCBI-DVG, and UniProt databases. These three datasets were merged into one usingIF techniques. This new dataset wascleaned and pre-processed by applying several criteria, e.g., eliminating preclinical assays that do not register values in biological activities. Next, we calculated the Sh(syst)_k_ of the different sub-systems involved, such as, drugs, protein sequences, genes and chromosomes using Markov Chains models. After that, PTOs with the form of MAs were used to quantify deviations in the structural parameters Sh(syst)_k_ (numerical parameters) concerning changes in the experimental conditions (categorical variables). This allowed us to quantify it in simple PTOs information from the structure and experimental conditions of assays of all the sub-systems involved. Finally, we trained, validated, and compared the IFPTML models. The role of the different sources of information was discussed as well. This kind of analysis opens a new way to carry IF combined with ML modeling towards discovering new antimalarial compounds using preclinical assays and proteome information.

## 2. Results

We developed various IFPTML models using PTOs and the MMAs operators [14]. The model calculated the scoring function *f*(v_ij_)_calc_ for outcome of i^th^ drug vs. j^th^ protein in preclinical assay multiple conditions of assay defined by the categorical variables **c**_j_. The first model developed was the IFPTML-GDA linear model. The Equation (1) of this model is the following:

(1) $$f{\left(v_{\mathrm{ij}}\right)}_{\mathrm{calc}}=-20.12298+99.13885\cdot f{\left(v_{\mathrm{ij}}\right)}_{\mathrm{ref}}\;\;\;\;\;\;+0.74880\cdot \Delta \mathrm{Sh}{\left(\mathrm{Drug};\mathrm{Csat}\right)}_{5c_{\mathrm{aj}}}\;\;\;\;\;\;\;-2.20919\cdot \Delta \mathrm{Sh}{\left(\mathrm{Drug};\mathrm{Hetero}\right)}_{5c_{\mathrm{aj}}}\;\;\;\;\;\;+3.36764\cdot \Delta \mathrm{Sh}{\left(\mathrm{Drug};\mathrm{Hx}\right)}_{1c_{\mathrm{aj}}}\;\;\;\;\;\;+2.39122\cdot \Delta \mathrm{Sh}{\left(\mathrm{Drug};\mathrm{Csat}\right)}_{1c_{\mathrm{pj}}}\;\;\;\;\;\;\;+2.25745\cdot \Delta \mathrm{Sh}{\left(\mathrm{Drug};\mathrm{Hetero}\right)}_{4c_{\mathrm{pj}}}\;\;\;\;\;\;\;-3.32408\cdot \Delta \mathrm{Sh}{\left(\mathrm{Drug};\mathrm{Hx}\right)}_{4c_{\mathrm{pj}}}\;\;\;\;\;\;\;-2.88041\cdot \Delta \mathrm{Sh}{\left(\mathrm{Drug};\mathrm{Csat}\right)}_{1c_{\mathrm{dj}}}\;\;\;\;\;\;\;+6.57931\cdot \Delta \mathrm{Sh}{\left(\mathrm{Drug};\mathrm{Halog}\right)}_{1c_{\mathrm{dj}}}\;\;\;\;\;\;\;-6.84622\cdot \Delta \mathrm{Sh}{\left(\mathrm{Drug};\mathrm{Halog}\right)}_{2c_{\mathrm{dj}}}\;\;\;\;\;\;\;-0.00877\cdot \Delta \mathrm{Sh}{\left(\mathrm{Chr};\mathrm{Gen}\right)}_{5c_{\mathrm{aj}}}\;\;\;\;\;\;\;+0.46021\cdot \Delta \mathrm{Sh}{\left(\mathrm{Prot};\mathrm{Seq}\right)}_{5c_{\mathrm{dj}}}\;\;\;\;\;\;\;\;\;\;\;\;\;\;\;\;n=17758\chi^{2}=6595.853p<0.05$$

The variables in this IFPTML model result from several procedures of pre-processing and post-processing (after obtaining the model) of the input/output variables. For instance, the output of the model is the scoring function *f*(v_ij_)_calc_. This is a real value function useful to quantify the possibilities with which the i^th^ drug gives a positive outcome in the j^th^ with preclinical assay with categorical variables c_j_ = c_aj_, c_pj_ and c_dj_ (experimental conditions, etc.).

**Figure 2:**
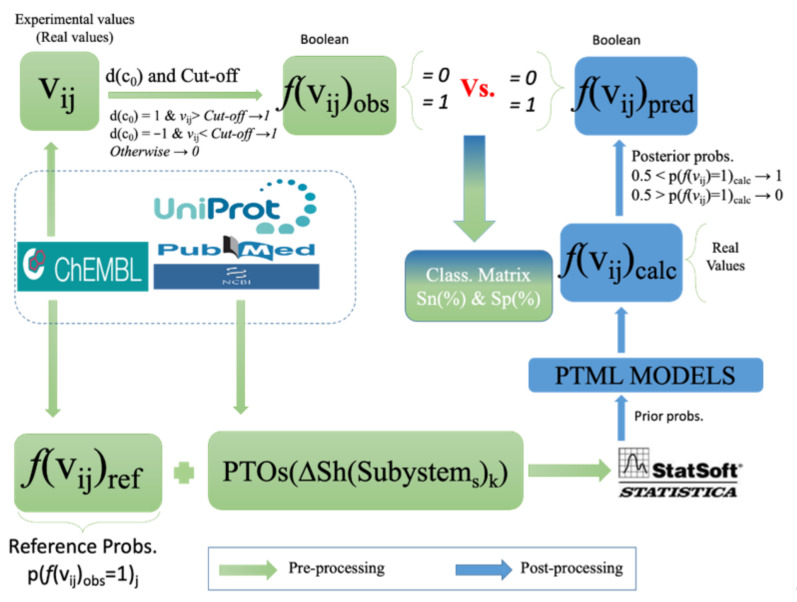
Variables pre-processing vs. post-processing.

In Figure 2, we give details of the procedures carried out for pre-processing and post-processing of the variables. After the post-processing procedure, we were able to compare inputs vs. outputs of the IFPTML model in order to obtain the classification matrix and measure its performance.

In addition in Table 1, we can see that the model is unbalanced with high values of Sp(%) and Accuracy Ac(%) > 98 in training and validation, but the values of Sn(%) are low. The other statistical parameters of the model are as follows: n is the number of cases used to train the model equal to 17,758;χ^2^ is the Chi-square statistics equal to 6595.853; and p is the p-level with a value less than 0.05. Multiple input variable encoding information related to the structure and conditions of assay of the drug is entered into the model using a forward stepwise feature selection strategy [30]. The model also includes variables encoding information about the protein sequence, gene sequence, and chromosome structure such as ΔSh(Prot; Seq)_5**c**dj_ andΔSh(Chr; Gen)_5**c**aj_.However, they seem to have a lower contribution.

In the classification matrix, we can see that the number of positive cases n(*f*(v_ij_) = 1) obtained after application of the cutoff values is very unbalanced with respect to the number of cases n(*f*(v_ij_) = 0) in the control series. In fact, we have n(*f*(v_ij_) = 1) = 232 in training and 74 in validation vs. n(*f*(v_ij_) = 0) = 13,087 in training and 4365 in validation for the control group. We carried out a cutoff scanning study to verify whether it could be caused due to a very restrictive value of the cutoffs or not. As can be seen in Table 2, the number of numbers of positive cases n(*f*(v_ij_) = 1) do not vary notably and is in all very low cases for all the ranges of cutoff which is interesting for antimicrobial chemotherapy uses. For instance, in the case of Inhibition(%) the n(*f*(v_ij_) = 1) < 230 for all values of cutoff in the range Inhibition(%) = 75–100. The number of positive cases increases in the range n(*f*(v_ij_) = 1) = 300–9700 only for Inhibition(%) <50%, which is not a clinically useful range. In other properties like IC_50_ (nM) and K_i_ (nM), the number of positive cases n(*f*(v_ij_) = 1) < 140, cases in all the cutoff 1–100 nM ranges and for all values of cutoff in the range Inhibition(%) = 75–100. Due to all these problems, we tried toalso test non-linear IFPTML models (see next section).

**Figure 3:**
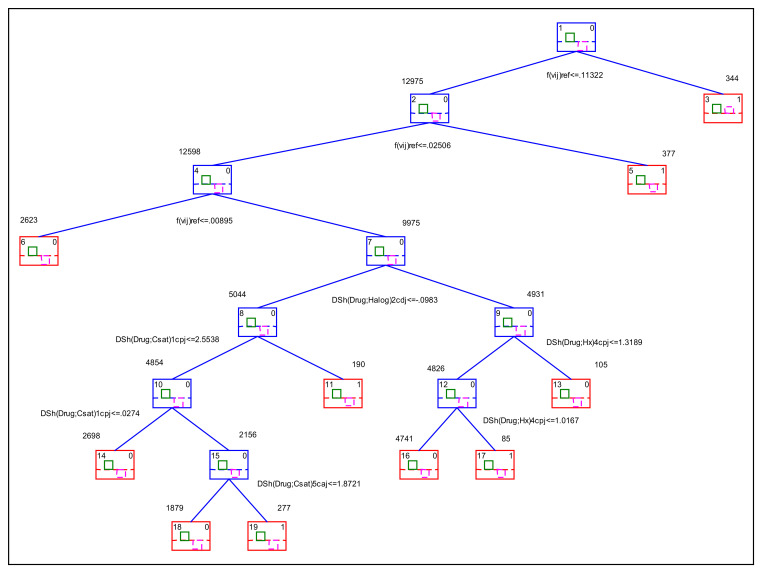
IFPTML-CTUS model decision tree.

One of the non-linear IFPTML models found was the Classification Tree (CT)—IFPTML model (IFPTML-CTUS), which is a CT model based on a Univariate Splitting (US) rule [30]. In this model, the prior probabilities with which a compound is predicted as active were set at π_1_ = 0.5. These probabilities are perfectly balanced compared with the unbalanced prior probabilities of π_1_ = 0.7 used in the GDA-IFPTML model. In Figure 3, we show the decision tree for the IFPTML-CTUS model. 

In Table 3, we show the results and coefficients of all the variables in the different splitting rules about the classification tree of this model. The variables that were entered into the model are ΔSh_1_ = ΔSh(Drug;Halog)_2_c_dj_, ΔSh_2_ = ΔSh(Drug;Csat)_1_c_pj_, ΔSh_3_ = ΔSh(Drug;Hx)_4_c_pj_, ΔSh_4_ = ΔSh(Drug;Csat)_1_c_pj_, ΔSh_5_ = ΔSh(Drug;Hx)_4_c_pj_, Sh_6_ = ΔSh(Drug;Csat)_5_c_aj_.

**Figure 4:**
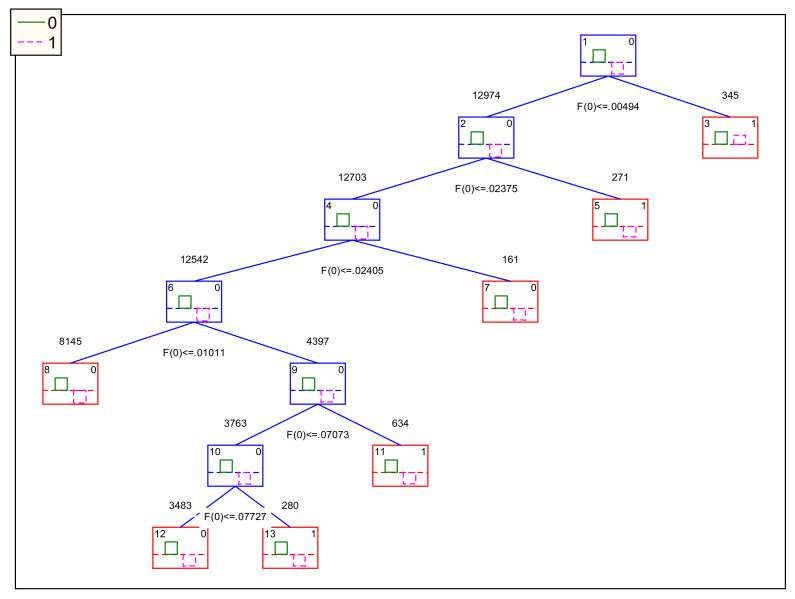
IFPTML-CTLC model decision tree.

Another model found was the IFPTML-CTLC, which is a IFPTML model based on CT but using Linear Combinations (LC) as split rules. In Figure 4, we show the decision tree for the IFPTML-CTLC model. In Table 4, we show the coefficients of all the variables in the different LCs used as splitting rules.

In the first instance, we compared the models in terms of performance. In Table 5, we can see a comparison of the three IFPTML models developed in this research: GDA, CTUS, and CTLC. The IFPTML-GDA model showed the lowest value of Sn(%) = 65.9/66.2 and Sp(%) = 98.7/98.8 for training and validation, respectively. Both IFPTML-CT models have balanced prior probabilities π_1_ = 0.5 with which a compound is predicted as active (compared π_0_ = 0.5). These values are perfectly equilibrated, remember that the IFPTML-GDA models presents important unbalance in this regard with π_1_ = 0.7 (compared π_0_ = 0.3). In addition, both IFPTML-CT models achieved values of Sn (%) and Sp(%) greater than 80.0%. The values of IFPTML-CTUS are equal to Sn (%) = 81.0/82.4 and Sp(%) = 91.7/91.6. The IFPTML-CTLCalso has high values of Sn (%) = 83.6/85.1 and Sp(%) = 89.7/89.8.

Next, we would like to compare the models in terms of number of input variables, LCs, and number of splitting rules. The IFPTML-GDA uses >10 input variables but only one LC with one splitting rule. Interestingly, the IFPTML-CTUS model uses 5 input variables and 9 splitting constants without relying upon the use of LCs. Conversely, the IFPTML-CTLC is by large the more complicated model of the three with >10 input variables and 6 LCs, each one with its respective splitting constants. For instance, it includes information about the sequence of the protein in the variable ΔSh(Prot;Seq)_5_**c**_dj_ and information about the gene and chromosome of this protein with the variable ΔSh(Chr;Gen)_5_**c**_aj_. According to these results, we can say that the last model is the best selection in terms of performance and inclusion of biologically relevant information.

Last, we should compare the models regarding the relevance of the biological information included in the input variables. The IFPTML-GDA model contains relevant information about drug structure, protein sequence, etc. By the contrary, the IFPTML-CTUS model does not include information about protein sequence, gene sequence, or chromosome structure. The missing information about the sequence of the protein invalidates the IFPTML-CTUS model for practical uses in the prediction of Antimalarial drugs against a protein target with specific sequence changes (mutations). In fact, mutations in the Malaria gene have been found to be important in the development of drug resistance mechanisms [31,32]. Lastly, the IFPTML-CTLC model includes biological relevant variables related to the target protein, etc., as well as the IFPTML-GDA model. Overall, the IFPTML-CTLC model is the most complex, but at the same time seems to be the more valuable because it is balanced, has high values of Sn(%) and Sp(%), and includes relevant biological information.

## 3. Discussion

### 3.1. IFPTML Linear Model with Multi-Condition Combinatorial Moving Averages (MMAs)

In order to evaluate the performance of the model in terms of Specificity Sp(%) and Sensitivity Sn(%), IFPTML-GDA transforms *f*(v_ij_)_calc_ into the Boolean variable *f*(v_ij_)_pred_. The variable *f*(v_ij_)_pred_ = 1 when the compounds arepredicted to be active in this assay; *f*(v_ij_)_pred_ = 0 otherwise. This variable gets the value *f*(v_ij_)_pred_ = 1 when the posterior probability with the compound is active p(*f*(v_ij_) = 1) ≥ 0.5. The IFPTML-GDA algorithm can estimate the values of posterior probabilities as a sigmoidal function p(*f*(v_ij_) = 1) = π_1_/(π_1_ + π_0_·Exp(-*f*(v_ij_)_calc_) of the prior probabilities π_1_ and π_0_ and the values of the score function.In this model, the prior probabilities with which a compound is predicted as active have been set π_1_ = 0.7 [30]. The deficient number of active compounds in ChEMBL datasetsomehow justifies this relatively high value of prior probability, see next discussion. 

The main advantage of this IFPTML algorithm is the obtention of a single global model. It means that a unified model has been constructed for preclinical assay optimization of new antimalarial compounds vs. the 28 protein sequences in many different assay conditions c_j_. In fact, the modelproperly predicts the outcome of 17,758 assays in total. This model will also be able to predict new antimalarial compounds for new protein sequences not included in the previous dataset. Otherwise, if we construct one model for each target protein, we will need to train/validate one model for each protein. It means, we need to train/validate a total of 28 individual models, excluding all other variable conditions. Consequently, the IFPTML algorithm can fit one model, performing the job of 28 classic models. In addition, each classic model must be trained with a smaller number of assays. In closing, the models for a single protein are unable to predict the results of one compound for other proteins and/or protein mutants, as they are not sequence sensible.

### 3.2. IFPTML-CTUS and IFPTML-CTLC Models

The models made the main emphasis on input variables related to chemical information about the structure of the drug and the conditions of assays.

### 3.3. IFPTML-CTLC Model Practical Use Example

**Figure 5:**
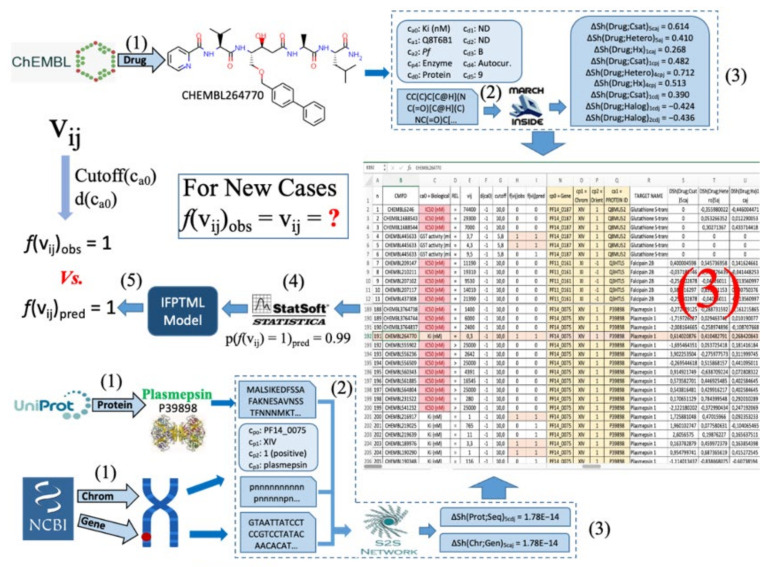
An example of the IFPTML-CTLC model.

In this section, we illustrate the use of the model with a practical example. We selected the molecule with code CHEMBL264770. See details about this compound in the Supplementary Materials. In Figure 5, we graphically depict all the steps necessary for processing a known or new compound with the present model using CHEMBL264770 as an example. In this figure, we illustrate the three main stages of the algorithm and their more important steps. The IF stage involves steps (1) and (2), the PT stage includes only step (3), and the ML stage includessteps (4) and (5). In step (1), all known information about molecule, target protein, gen, chromosome, and/or assay conditionsis downloaded from three databases ChEMBL, UniProt, and NCBI-GDV. In the case of a new compound, the value of biological activity v_ij_ is unknown, but we know all other information about the assay. This information includes numerical variables andcategorical variables that encode information on the experimental conditions of the preclinical trials or on the nature and quality of the data. For the molecule CHEMBL264770, the activity parameter is Ki (nM), the Uniprotaccession ID of target protein is P39898, the assay organism is *Plasmodium falciparum*, the ChEMBL function is Enzyme, the target mapping is a protein, the APD*’*s name and confidence are labeled as ND (Not data), the assay type is B, the curated by Autocur, the number of Confidence Score is 9, and Canonical SMILES. Other data downloaded from NCBI-GDV database are the biological information about target proteins, genes, and chromosomes. Thus, for this example the name of gene in the chromosome XIV is *PF14_0075*, the orientation of gene is 1 which means positive, the protein function is plasmepsin, the nucleotides recurrence of gene and the Genes orientationsin thischromosome. All the information downloaded from these databases was copied into an .xlsx file. In step (2), we calculated the Shannon entropies of the drugs, protein sequences, and chromosome in order to quantify the structural information. For inputs, we used the Canonical SMILES of drugs, the sequence of proteins, sequence of gene, and gene orientation networks (GOIN) of chromosomes. The software MARCH-INSIDE was used to calculate the Shannon information entropy of drugs Sh(drug). Other variables calculated werethe Shannon entropies of Amino Acids recurrence Sh(prot), Nucleotides recurrence Sh(gene), and Gene orientation in the chromosome Sh(Chr). These variables werecalculated using the S2SNetwork tool. After step (2) we finished the IF phase and entered the PT phase. In step (3), we calculated PTOs with the form of Moving Average (MA) operators. Up to this point, data cleaning and pre-processing hadbeen performed together with the calculations of the operators applying Perturbation Theory. In step (4), we used the software STATISTICA to run different ML algorithms. For the new molecule, we substituted the values of the operators ΔSh(Drug_i_)_k,**c**aj_, ΔSh (Prot_i_)_k,**c**pj_, etc., into these models. Using the IFPTML-GDA modelfor instance, we can predict an output of p(*f*(v_ij_)=1) = 0.99 for this example. This means that the model predicts that this compound is expected to have a value K_i_< 10 nM (cut-off) with a probability of 0.99. Finally in step (5), we can conclude that the *f*(v_ij_)_pred_ = 1 (the compound can be considered active according to this assay). As this compound is already known, we can corroborate that this prediction coincides with the observed classification *f*(v_ij_)_obs_ = 1 which comes from a real value of Ki = 0.3 nM. In the case of a compound not previously assayed, one would need to assay the compound in order to corroborate this prediction.

## 4. Materials and Methods

### 4.1. ChEMBL Dataset

We downloaded all the information about proteins and unique assays only for *Pf.* The dataset does not contain another species of intracellular protozoa of the genus *Plasmodium*. The dataset was obtainedfrom the ChEMBL database (https://www.ebi.ac.uk/chembl/g/#browse/targets (accessed on 15 November 2018)) using the browser targets tool [33,34,35,36]. Initially, the total proteins registered in ChEMBL was 33 for *Pf*. However, the total was 28 proteins, after performing the data pre-processing, which is explained in detail in the next section. The proteins werecategorized as follows: 21 Enzymes, 3 Transporters, 1 Epigenetic Regulator, 3 Others Cytosolic Proteins, and 5 Unclassified Proteins. The total number of unique assays outcomes (endpoints) registered for the 33 proteins was 18,381 (statistical cases). Each protein category contains mainly the following fields: ChEMBLID, Preferred Name, UniProt Accession (used to obtain the protein sequences in the UniProt Database), and other fields such as: Target Type, Organism, Compounds, and Endpoints, also called Bioactivities (used to obtain the different assays in the ChEMBL Database). For example, an enzyme ChEMBLID = “CHEMBL1697656” was registeredwith its Preferred Name = “Glutathione S-transferase”,UniProt Accession = “Q8MU52”, Target Type = “Single Protein”, Organism = “*Plasmodium falciparum*”, Compounds = “4”, and Endpoints = “6”. Additionally, each endpoint comes from a unique assay with the following main fields: CMPD ChEMBLID, Molecule Name, SMILES, Activity ID, Standard Type, Relation, Standard Value, and Standard Units. Other fields are Assay ID, Assay ChEMBLID, Assay Type, Description, Protein Accession (UniProt Accession), Journal, Year, Volume, and Issue, among others.

### 4.2. NCBI-GDV Dataset

The *Pf* genome used was originally reported in the Mapviewer database [7,8]. Currently, this dataset is available in the new NCBI-GDV database (https://www.ncbi.nlm.nih.gov/genome/gdv/ (accessed on 15 November 2017)) [8]. Initially, the *Pf* genome had 14 different chromosomes. Each chromosome contains an average of 383 genes. In this work, we used only 10 out of these 14 chromosomes because the proteins codified by the remnant 4 chromosomes have no biological assays reported in ChEMBL. The genes have a start-and-stop position within the chromosome. The database reports the position (P_ik_) of each gene in the chromosome and a description of the biological function. The dataset registered the biological sequence of nucleotides of each gene. Additionally, the dataset reports the symbol, the orientation of the gene, as positive or negative (O_ik_= 1 or O_ik_ = −1). This information has been found to be somehow relevant to the biological activity of some proteins in *Pf* proteome. Consequently, in this work we also used the Chromosome Gene Orientations Inversion Networks (GOINs) of *Pf* proteome assembled with P_ik_ and O_ik_ information in a previous work [29].

### 4.3. UniProt Dataset

We downloaded the biological sequence of amino acids of the 28 proteins registered in ChEMBL in FASTA format. The dataset was obtained from UniProt database (https://www.uniprot.org/ (accessed on 15 November 2018)) using the browser protein tool [9,10,11]. In turn, the FASTA format has two parameters that were used in this work: string of characteristics and sequence of proteins.

### 4.4. ChEMBL, NCBI-GDV, and UniProt Information Fusion

We constructed a dataset based on the three previous datasets. In so doing, we carried out an IF process [37,38,39,40]. After performing the IF process, the working dataset created contained a total of 18,381 outcomes (rows). We added the canonical SMILE codes and their respective Shannon’s Entropy values for each chemical compound. The simplified molecular-input line-entry system (SMILES) codes downloaded from ChEMBLare a notation system used to codify information about the chemical structure of the compounds [41]. SMILES-like representations have been largely used in Cheminformatics [42,43,44,45,46,47]. We also aggregated the protein sequence and the Shannon’s Entropies in each row according to the respective Protein Accession ID. In addition, we added the parameters of each gene and the Shannon’s Entropy values for each protein.

### 4.5. Pre-Processing of the Working Dataset

**Figure 6:**
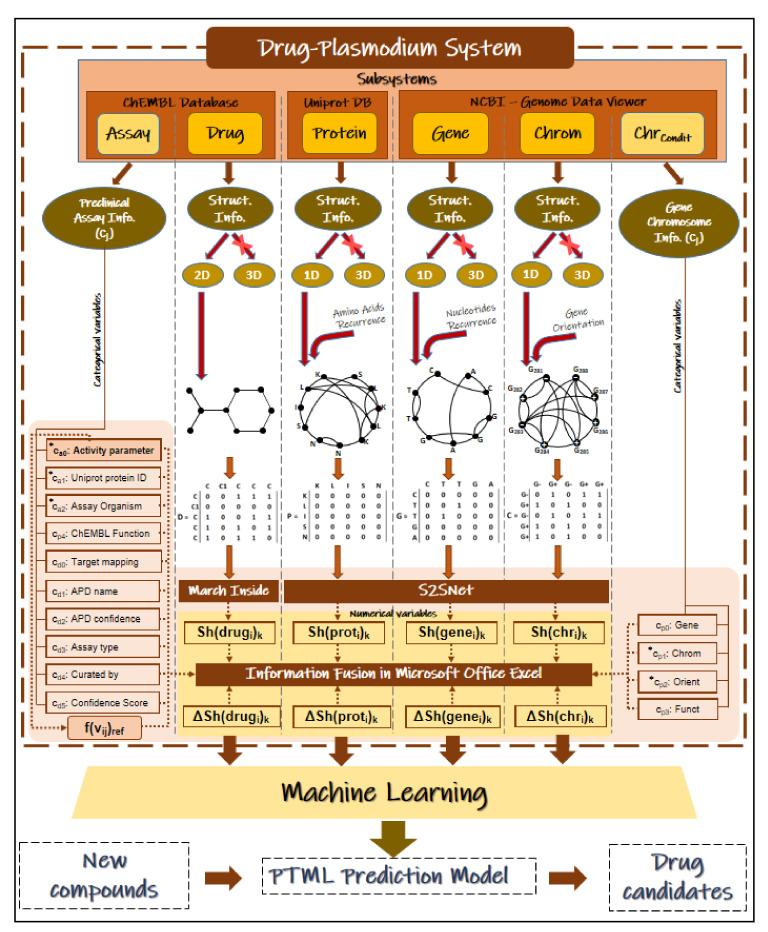
IFPTML model development and IF process.

Firstly, we deleted rows where no values were reported for the variables v_ij_, PSA, or AlogPin order toclean the dataset. For this reason, the categories of the variable c_p4_are reduced to 19 Enzymes, 2 Transporters, 1 Epigenetic Regulator, 2 Others Cytosolic Proteins, and 4 Unclassified Proteins. The total of proteins valid from ChEMBL were 28. Therefore, the data removed represents only a 3.4% of all working dataset. Moreover, all the empty cells of chain type were replaced with the tag ND (No Data). At the end, the dataset to obtain the IFPTML based model had 17,758 rows. In Figure 6, we illustrate the different steps given to pre-processing the data and carrying out the IF process.

### 4.6. IFPTML Shannon Information Theory Models

In Figure 6, we illustrate details of the different steps given to pre-processing the data and train/validate the IFPTML model. First, we performed the IF process, next we calculated the Sh(Subsystem_s_)_k_ values, the f(v_ij_)_ref_ function values, and the PTOs values (input variables), and then we proceeded to seek the IFPTML models. See more details about the calculation of input/output variables in the next sections. The objective of the IFPTML model is to predict a function f(v_ij_)_calc_ of the observed values f(v_ij_)_obs_. In order to develop the IFPTML model, we took into consideration both structural and functional information for the calculation of the input variables. The structural information refers to the chemical structure of the drug as well as structural features of the target protein, the gene encoding for this target protein, and chromosome of this gene.

We can approach the present problem from the point of view Shannon’s Information theory and the theory of Complex Systems. In this sense, we can quantify the relevant structural/functional information of the system with Sh(Syst)_k_ values calculated using a Markov Chain approach [28]. After that, we calculated the external property of the system *f*(v_ij_)_calc_ as a function of a value of reference *f*(v_ij_)_ref_ and a function *f*(Sh(Syst)_k,**c**j_) of the structural and functional information. In the Equation (2) we used an IFPTML additive approach to include and separate the different parts of the system or subsystems.

(2) $$f{\left(v_{\mathrm{ij}}\right)}_{\mathrm{calc}}=a_{0}+a_{1}\cdot f{\left(v_{\mathrm{ij}}\right)}_{\mathrm{ref}}+\overset{s_{\max},k_{\max,j_{\max}}}{\underset{s=0,k=0,j=0}{\sum }}a_{s,k,j}\cdot \mathrm{PTO}\left[\mathrm{Sh}{\left({\mathrm{Subsystem}}_{s}\right)}_{k,c_{j}}\right]$$

The function of reference *f*(v_ij_)_ref_ quantifies the expected value of probability of biological activity for a compound measure under certain experimental conditions specified by the partition **c**_j_of categorical variables. The subsystems considered are Subsystem_0_ = drug, Subsystem_1_ = protein, Subsystem_2_ = gene, and Subsystem_3_ = chromosome. The information about each subsystem will be quantified with the respective Shannon’s Entropy information measure values of order k^th^ for each subsystem Sh(Subsystem_s_)_k_. For instance, Sh(Subsystem_0_)_k_ = Sh(Drug)_k_ and Sh(Subsystem_1_)_k_ = Sh(Prot)_k_, etc. The value k^th^ can register values from 0 to 5. In addition, the IFPTML model uses PTOs to quantify the deviation (perturbations) in continuous variables (structural parameters, time, concentration, etc.) with respect to functional information encoded by categorical variables **c**_j_ (experimental conditions), see details in next sections [14].

In this context, in the Equation (3), we can illustrate the general form of an IFPTML model for the linear cases. In the Equation (4), we selected the linear cases for the sake of simplicity, but in this work, we also reported non-linear models. We can extend the previous equation of the model to write down a general form of the IFPTML model. In so doing, we used MMA as PTOs operators as follows.

(3) $$f{\left(v_{\mathrm{ij}}\right)}_{\mathrm{calc}}=a_{0}+a_{1}\cdot f{\left(v_{\mathrm{ij}}\right)}_{\mathrm{ref}}+\overset{s_{\max},k_{\max,j_{\max}}}{\underset{s=0,k=0,j=0}{\sum }}a_{s,k,j}\cdot \Delta \mathrm{Sh}{\left({\mathrm{Subsystem}}_{s}\right)}_{k,c_{j}}$$

(4) $$f{\left(v_{\mathrm{ij}}\right)}_{\mathrm{calc}}=a_{0}+a_{1}\cdot f{\left(v_{\mathrm{ij}}\right)}_{\mathrm{ref}}+\overset{k_{\max},j_{\max}}{\underset{k=0,j=0}{\sum }}a_{s,k,j}\cdot \Delta \mathrm{Sh}{\left(\mathrm{Drug}\right)}_{k,c_{j}}+\overset{k_{\max},j_{\max}}{\underset{k=0,j=0}{\sum }}a_{s,k,j}\cdot \Delta \mathrm{Sh}{\left(\mathrm{Prot}\right)}_{k,c_{j}}\;+\overset{k_{\max},j_{\max}}{\underset{k=0,j=0}{\sum }}a_{s,k,j}\cdot \Delta \mathrm{Sh}{\left(\mathrm{Gene}\right)}_{k,c_{j}}+\overset{k_{\max},j_{\max}}{\underset{k=0,j=0}{\sum }}a_{s,k,j}\cdot \Delta \mathrm{Sh}{\left(\mathrm{Chr}\right)}_{k,c_{j}}$$

### 4.7. Output Variable and Function of Reference

In this work, we developed a IFPTML model for the study of experimental values v_ij_ of biological activity of the i^th^ drug in j^th^ preclinical assays of Antimalarialdrugs reported in ChEMBL database. Due to the high number of different biological parameters with different scales and levels of errors, we discretized them to obtain the Boolean function *f*(v_ij_)_obs_ to develop a classification model. Firstly, we performed the pre-processing in order to clean the dataset, define/calculate the input, and output variables. Specifically, the *f*(v_ij_)_obs_and *f*(v_ij_)_ref_ values have been calculated using excel functions and added to the dataset, see Table 6. For instance, for the calculation of the number of cases with one specific level of c_a0_ (one specific parameter of biological activity) we used the function COUNTIF. The first argument in the syntax is Range(c_a0_) = cells that contain all the values of the categorical variable c_a0_ (names of the parameters of biological activity measured in each preclinical assay). The second argument is Criteria(c_a0_) = cells containing the value of one unique level of c_a0_ (name of one specific parameter of biological activity). The function runs over all Range(c_a0_) comparing Criteria(c_a0_) with the specific cell of the Range(c_a0_). Other arguments used in different functions are Range(v_ij_) = cells that contain all the values of biological activity for all preclinical assays (v_ij_), Units(c_a0_) = the units of the biological activity measured (c_a0_), desirability d(c_a0_) = 1 or −1, and Range(*f*(v_ij_)_obs_) = cells that contains the *f*(v_ij_)_obs_ value [14].

### 4.8. Shannon Entropy Measures

The previous IFPTML equations were inputted asSh(Subsystem_s_)_k_ variables. We calculated the Shannon’s Entropies values Sh(Drug)_k_, Sh(Prot)_k_, Sh(Gene)_k_, and Sh(Chrom)_k_ to quantify the structure information of the different subsystems. We used the tool MARkovCHains Invariants for Network Selection and DEsign (MARCH-INSIDE) to calculate the Sh(Drug)_k_ values of drugs [48]. The software MARCH-INSIDE was usedto input the Simplified Molecular Input Line Entry Specification (SMILES) codes for each compound downloaded from ChEMBL. On the other hand, we used the tool Sequences to Networks (S2SNet) [28] to calculate information index values Sh(Prot)_k_, Sh(Gene)_k_, and Sh(Chrom)_k_ about the sequence and recurrence of different amino acids into the proteins, nucleotides into the genes, and genes into the chromosomes. The software S2SNet was used to input the sequences of proteins and genes downloaded from UniProt and NCBI-GDV, respectively. S2SNet was also used to input a np (negative/positive) sequence code to express the orientation of reading and position of each gene into the chromosome.

**Figure 7:**
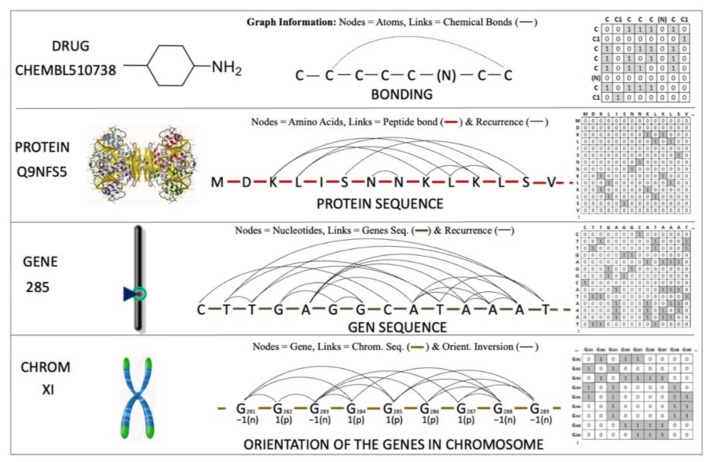
Illustration of different representations to represent multiple molecular systems.

Both MARCH-INSIDE (drugs) and S2SNet (proteins, genes, and chromosomes) use a graph to represent the parts of the subsystem (nodes) and the relationships (link) among them into the structure of the subsystem. The parts of the subsystems are atoms, amino acids, nucleotide bases, or genes. The links among them are chemical bonds, peptide bonds, gene sequence, or gene position according to the system. The S2SNet software also takes into account relationships of recurrence to specific types of amino acids, nucleotides, and gene orientation. Figure 7 illustrates some examples of the graphs used to represent the different subsystems. It shows the name, the representation graph, and a small part of the graph with its nodes and links. We can see in this figure, from bottom to top, the chromosome XI represented by genes and the links to the pairs of genes with inverse orientation. The graph’s nodes of gene 285 with its representation graph in the chromosome, and the graph with its nodes represented by the nucleotides and links represented by the gene sequence by their recurrences. The protein Q9NFSS has nodes to amino acids and links to peptide bonds and the recurrence. Finally, the graph of the CHEMBL510738 drug was representedwith atoms (nodes) and Chemical Bonds (links).

Both MARCH-INSIDE and S2SNet associates a node adjacency matrix **A**(Subsystem_s_) to the respective graphs to carry out a numerical representation of the system (see Figure 7). Next, both software transforms the adjacency matrix of each subsystem **A**(Subsystem_s_) into a Markov matrix Π_1_(Subsystem_s_), not represented in Figure 7. After that, both tools calculate the natural powers of order k^th^ for each matrix Π_1_(Subsystem_s_). Last, both software use the Chapman-Kolmogórov equations to calculate the absolute probabilities ^a^p(n/s)_k_ for each node in a given subsystem (n/s) [28,48]. With these probabilities and the Equation (5), the software performs the calculation of the different Sh(Drug)_k_, Sh(Prot)_k_, Sh(Gene)_k_, and Sh(Chrom)_k_ values.

(5) ShSubsystemsk=−∑n=1nmaxpan,sk ·log(pan,sk)

### 4.9. Partitions of Categorical Variables

We created two partitions (subsets) of categorical variables from ChEMBL dataset to encode all the functional or non-structural information. The first partition of categorical variables was **c**_assayj_ (abbreviated as **c**_aj_). The second partition was **c**_dataj_(abbreviated as **c**_dj_). These partitions contain variables that encode information about experimental conditions of preclinical assays (**c**_aj_) or about the nature and quality of data (**c**_dj_). These categorical variables include information about 22 biological activity types (c_a0_), 28 target proteins (c_a1_), and 9 organisms of the assay (c_a2_), etc. We also created another partition (**c**_protj_= **c**_pj_) including categorical variables with biological information about the target proteins, genes, and chromosomes. These variables cover32 genes (c_p0_), 10 chromosomes (c_p1_), gene orientation (c_p2_), and 31 protein functions (c_p3_). Table 7 depicts details of these partitions.

### 4.10. Perturbation-Theory Operators (PTOs)

As we mentioned before, the IFPTML model use PTOs to quantify the deviation (perturbations) in continuous variables (structural parameters, time, concentration, etc.) with respect to functional information encoded by categorical variables **c**_j_ (experimental conditions). In this work we selected the MMAs operators of type PTO(Sh(Subsystem_s_)_k_ = ΔSh(Subsystem_s_)_k,**c**j_ = Sh(Subsystem_1_)_k_−<Sh(Subsystem_1_)_k,**c**j_> or *f*(Sh(Subsystem_s_)_k_ = ΔSh(Subsystem_s_)_k,**c**j_ = Sh(Subsystem_1_)_k_−<Sh(Subsystem_1_)_k,**c**j_>. These operators quantify the deviation (gain or loss in information) of the specific value Sh(Subsystem_1_)_k_ of the subsystem concerning the average <Sh(Subsystem_1_)_k,**c**j_> (expected value) of information for all cases measured under the same experimental conditions. We used three different partitions **c**_j_ of categorical variables to codify the experimental conditions and/or non-structural information (see next section). Moreover, in this data pre-processing stage, we have calculated the PT operators similar to Box-Jenkins MA operators that are used asinput data. In this context, **c** (with **c** in boldface) refers to a vector of multiple combinations of categorical variables at the same time. The partitions of the categorical variables used here are **c**_assayj_, **c**_protj_, and **c**_dataj_. These partitions are fusions of categorical variables related to the pharmacological assay (**c**_assayj_), the nature of the drug target (**c**_protj_), or about the nature and/or accuracy of the data measured (**c**_dataj_). For simplicity’ssake, we abbreviate these partitions as **c**_assayj_ = **c**_aj_, **c**_protj_ = **c**_pj_, and **c**_dataj_ = **c**_dj_. The partition**c**_aj_ = (c_a0_,c_a1_, c_a2_) included the following categorical variables: biological activity (c_a0_), the UniProt protein accession ID (c_a1_), and the organism of assay (c_a2_). In the Supplementary Materials we detailed all fused datasets of drugs, unique sequences, proteins, chromosomes, genes, Shannon Entropies values, and the PTO’s values, this process is called the IF technique. Table 8 shows details of the Perturbation-Theory Operators.

### 4.11. IFPTMLModel Training and Validation

The first step to develop the IFPTML models [12,13,14,15,16,17] was to download all the information about preclinical assays, drugs structure, protein sequences, gene sequences, and chromosomes information from public databases (ChEMBL, UniProt, NCBI-GDV**).** The second step was to carry out a pre-processing of all the previous information in order to calculate the*f*(v_ij_)_obs_ (dependent variable) and *f*(v_ij_)_ref_.Next, we calculated the Sh(Subsystem_s_)_k_ values (input variables). This includes a process of information fusion including data from the different databases (ChEMBL, UniProt, NCBI-GDV). Once data have been prepared for analysis, wethen run the ML algorithms General Discriminant Analysis (GDA), Classification Tree (CT) with Univariate Splits (CTUS), and CT with Linear Combination (CTLC) to seek alternative IFPTML models. All the IFPTML models were developed using STATISTICA [30] software v. 12.

## 5. Conclusions

Computational prediction of new Antimalarial compounds is a very important goal for the pharmaceutical industry. However, the huge amount of information available from different sources makes the analysis of data for the discovery of new compoundsdifficult. The IFPTML method allowed us to conduct the fusion and analysis of three different datasets from the databases ChEMBL, UniProt, and NCBI-GDV to achieve this goal. The ChEMBL dataset contains outcomes for17,758unique assays including numeric descriptors (variables) for the structure of compounds. The IFPTML algorithm was successful in accounting for both numerical information (structural parameters) and categorical information (multiple experimental conditions) of the three datasets. Shannon’s entropy measures Sh_k_ (numerical variables) were useful to quantify the information about the structure of drugs, protein sequences, gene sequences, and chromosomes. In addition, MMAs of different partitions of categorical variables from categorical variables from theChEMBL dataset were useful to encode multiple experimental conditions of preclinical assays and information about targets proteins, genes, and chromosomes. The IFPTML-CTLC model is the most complex in terms of number of input variables, number of LCs, and number of splitting rules. However, the IFPTML-CTLC model showed better performance than the IFPTML-GDA and includes more biologically relevant information than the IFPTML-CTUS model. This model could become a useful tool for the optimization of pre-clinical assays of new Antimalarial compounds taking into consideration the structure of the drug, the specie of *Plasmodium*, the sequence of the target protein, and other multiple parameters.

## Figures and Tables

**Table 1:** IFPTML-GDA model result.

Observed	Statistical	Predicted	Predicted Sets		
Sets^a^	Parameter^b^	Statistics	n_j_	*f*(v_ij_)_pred_ = 0	*f*(v_ij_)_pred_ = 1
Training Series					
*f*(v_ij_)_obs_= 0	Sp(%)	98.8	13,087	12,934	153
*f*(v_ij_)_obs_ = 1	Sn(%)	65.9	232	79	153
total	Ac(%)	98.3	13,319		
External Validation Series					
*f*(v_ij_)_obs_= 0	Sp(%)	98.7	4365	4310	55
*f*(v_ij_)_obs_ = 1	Sn(%)	66.2	74	25	49
total	Ac(%)	98.2	4439		

^a^The observed classification classes are two: drugs with a desired level of biological effect observed *f*(v_ij_)_obs_= 1 or *f*(v_ij_)_obs_= 0 otherwise. ^b^ Sn (%) = Sensitivity, Sp (%) = Specificity and AC (%) = Accuracy.

**Table 2:** Selected values of multi-condition averages for different combinations of assay conditions.

c_0_ = Activity (Units)	Cut-off(c_0_)								Total
	1	10	25	50	75	95	100	200	
Inhibition (%)	9785	1535	564	376	228	78	39	-	13,469
IC_50_ (nM)	2	29	49	81	101	108	110	133	3715
K_i_ (nM)	24	78	100	120	132	134	138	160	369
Other Activities	59	133	146	148	150	149	150	152	205
n(*f*(v_ij_)=1)	9870	1775	859	725	611	469	437	445	17,758
n(*f*(v_ij_)=0)	7888	15,983	16,899	17,033	17,147	17,289	17,321	17,313	

**Table 3:** IFPTML-CTUS model coefficients.

Class	Left	Right	Control	Active	Predict.	Split	Split
Node	Branch	Branch	n(f(v_ij_) = 0)	n(f(v_ij_) = 1)	Class	Constant	Variable
1	2	3	13,087	232	0	0.11321607	*f*(v_ij_)_refi_
2	4	5	12,903	72	0	0.02505894	*f*(v_ij_)_refi_
3			184	160	1		--
4	6	7	12,542	56	0	0.00895431	*f*(v_ij_)_refi_
5			361	16	1		--
6			2623	0	0		--
7	8	9	9919	56	0	−0.0982586	ΔSh(Drug;Halog)_2_c_dj_
8	10	11	5006	38	0	2.55375728	ΔSh(Drug;Csat)_1_c_pj_
9	12	13	4913	18	0	1.318866	ΔSh(Drug;Hx)_4_c_pj_
10	14	15	4821	33	0	0.02739699	ΔSh(Drug;Csat)_1_c_pj_
11			185	5	1		--
12	16	17	4809	17	0	1.01671015	ΔSh(Drug;Hx)_4_c_pj_
13			104	1	0		--
14			2681	17	0		--
15	18	19	2140	16	0	1.87205633	ΔSh(Drug;Csat)_5_c_aj_
16			4726	15	0		--
17			83	2	1		--
18			1868	11	0		--
19			272	5	1		--

**Table 4:** IFPTML-CTLC model coefficients.

Var	Coeff.	f(v_ij_)_01_	f(v_ij_)_02_	f(v_ij_)_03_	f(v_ij_)_04_	f(v_ij_)_05_	f(v_ij_)_06_	Mean	S.D.
Split const.	a_00_	−0.005	−0.024	−0.024	−0.010	−0.071	−0.077	−0.04	0.03
*f*(v_ij_)_ref_	a_01_	0.044	0.762	0.751	0.818	2.678	2.881	1.32	1.17
ΔSh(Drug;Csat)_5_c_aj_	a_02_	0.000	0.008	−0.001	−0.003	−0.008	−0.007	0.00	0.01
ΔSh(Drug;Hetero)_5_c_aj_	a_03_	−0.001	−0.010	−0.042	−0.033	−0.103	−0.143	−0.06	0.06
ΔSh(Drug;Hx)_1_c_aj_	a_04_	0.001	0.020	0.047	0.047	0.120	0.160	0.07	0.06
ΔSh(Drug;Csat)_1_c_pj_	a_05_	0.001	0.014	0.020	0.023	0.083	0.093	0.04	0.04
ΔSh(Drug;Hetero)_4_c_pj_	a_06_	0.001	0.009	0.036	0.028	0.078	0.109	0.04	0.04
ΔSh(Drug;Hx)_4_c_pj_	a_07_	−0.001	−0.017	−0.038	−0.037	−0.092	−0.117	−0.05	0.04
ΔSh(Drug;Csat)_1_c_dj_	a_08_	−0.001	−0.019	−0.016	−0.017	−0.065	−0.079	−0.03	0.03
ΔSh(Drug;Halog)_1_c_dj_	a_09_	0.003	0.057	0.087	0.088	0.713	0.577	0.25	0.31
ΔSh(Drug;Halog)_2_c_dj_	a_10_	−0.003	−0.059	−0.094	−0.095	−0.740	−0.609	−0.27	0.32
ΔSh(Chr;Gen)_5_c_aj_	a_11_	0.000	0.000	0.002	0.002	0.039	0.075	0.02	0.03
ΔSh(Prot;Seq)_5_c_dj_	a_12_	0.000	0.004	−0.002	−0.003	0.008	0.024	0.01	0.01

**Table 5:** Comparison of models with different algorithms.

Algorithm	Set	Class	Stat Param.	Value (%)	f(v_ij_)_pred_ = 0	f(v_ij_)_pred_ = 1
IFPTML GDA π_0_ = 0.30 π_1_ = 0.70	Train	*f*(v_ij_)_obs_ = 0	Sp	98.8	12,934	153
		*f*(v_ij_)_obs_ = 1	Sn	65.9	79	153
	Validation	*f*(v_ij_)_obs_ = 0	Sp	98.7	4310	55
		*f*(v_ij_)_obs_ = 1	Sn	66.2	25	49
IFPTML CTUS π_0_ = 0.50 π_1_ = 0.50	Train	*f*(v_ij_)_obs_ = 0	Sp	91.7	12,002	1085
		*f*(v_ij_)_obs_ = 1	Sn	81.0	44	188
	Validation	*f*(v_ij_)_obs_ = 0	Sp	91.6	3997	368
		*f*(v_ij_)_obs_ = 1	Sn	82.4	13	61
	Train	*f*(v_ij_)_obs_ = 0	Sp	89.8	11,751	1336
IFPTML CTLC		*f*(v_ij_)_obs_ = 1	Sn	83.6	38	194
π_0_ = 0.50 π_1_ = 0.50	Validation	*f*(v_ij_)_obs_ = 0	Sp	89.7	3917	448
		*f*(v_ij_)_obs_ = 1	Sn	85.1	11	63

**Table 6:** More relevant functions used in the data pre-processing stage.

Variable	Excel Functions Syntax	Notes
n_j_(c_a0_)	=COUNTIF(Range(c_a0_), Criteria(c_a0_))	Function that determines the total number of cases for each Biological activity in the dataset.
<v_ij_(c_a0_)>	=AVERAGEIF (Range(c_a0_), Criteria(c_a0_), Range(v_ij_))	Calculates the average of all the standard values of biological activity in the dataset. It is used as an argument for the cutoff(c_a0_) function.
cutoff(c_a0_)	=IF(Units(c_a0_) = %, 95, IF(Units(c_a0_) = nM, 10, <v_ij_(c_a0_)>)	The cutoff value is used to decide if the compounds is active or not. For the values of Activity(%) and Inhibition(%), the cutoff(c_a0_) = 95%.Similarly, for the IC_50_(nM), K_i_(nM), and K_m_(nM), the cutoff(c_a0_) = 10 nM, etc.
d(c_a0_)	=OR(d(c_a0_) = 1, d(c_a0_) = −1)	Indicates that the measured parameter increases or decreases directly with a desired or not desired biological effect.
*f*(v_ij_)_obs_	=IF(AND(v_ij_> cutoff(c_a0_), d(c_a0_) = 1), 1, IF(AND(v_ij_ ≤ cutoff(c_a0_), d(c_a0_) = −1), 1, 0))	*f*(v_ij_)_obs_ = 1 for active compounds or *f*(v_ij_)_obs_ = 0 for control group according to the set of cutoff and desirability values used for each c_a0_. It is the function used as output to train the IFPTML model.
n(*f*(v_ij_) =1)	=COUNTIF(Range(c_a0_), Criteria(c_a0_), Range(*f*(v_ij_)_obs_, 1))	Function that determines the total number of each Biological activity in the dataset and f(v_ij_)_obs_ equal to 1.
*f*(v_ij_)_ref_	=n(*f*(v_ij_)=1)/n_j_(c_a0_)	The function of reference *f*(v_ij_)_ref_ = p(*f*(v_ij_)=1/c_a0_) is the probability with which the observed function gets the value *f*(v_ij_)_obs_ = 1, positive assay. It is used as the first input variable of the IFPTML model.

**Table 7:** Partitions and levels (unique values) taken by the categorical (not ordered) input variables.

Partition (c_j_)	Var.	Information	NL^a^	Unique Levels
c_assayj_ (c_aj_)	c_a0_	Biological activity	22	Inhibition(%); IC_50_(nM); K_i_(nM); IC_50_(ug.mL^−1^); BHIA_50_(-); IC_50_(mill equivalent); FC(-); K_inact_(/min); Activity(%); VAR(-); Ratio(-); Ratio(/M/s); IC_50_(molar ratio); Ratio IC_50_(-); Mean(pM mg^−1^); GST activity (mU mg^−1^); K_m_(nM); Ratio(/s/M); Activity(-); K_a_(10^3^/M/s); K_cat_(/s); Inhibition(uM)
	c_a1_	UniProt protein accession ID	28	Q8MU52; Q3HTL5; Q9NBA7; Q9NFS5; Q8T6J6; Q25856; P39898; Q9N6S8; Q0PJ46; Q6T755; Q8MMZ4; Q868D6; Q25917; Q9GSW0; Q9NAW4; O77078; Q9NAW2; Q9BJJ9; Q8T6B1; Q9N623; Q9XYC7; P05227; P11144; Q17SB2; O77239; Q9Y006; O96214; O97467
	c_a2_	Assay Organism	9	*Plasmodium falciparum*; *Plasmodium falciparum* K1; *Plasmodium falciparum* NF54; *Plasmodium falciparum* Dd2; *Plasmodium* sp.; *Plasmodium yoelii*; *Plasmodium berghei*; *Leishmania Mexicana*; ND (No registered data)
c_dataj_ (c_dj_)	c_d0_	Target mapping	2	Protein; Homologous protein
	c_d1_	APD name	9	Peptidase C1; Pkinase; Peptidase S8; Asp; OMPdecase; Spermine synth; Sugar tr; Hist deacetyl
	c_d2_	APD confidence	2	ND (No-Data); high
	c_d3_	Assay type	2	Binding (B) = Data measuring binding of compound to a molecular target.Functional (F) = Data measuring the biological effect of a compound.
	c_d4_	Data curation level	3	Autocuration; Intermediate; Expert
	c_d5_	Confidence score	2	8 = Homologous single protein target assigned. 9 = Direct single protein target assigned
c_protj_ (c_pj_)	c_p0_	Gene	32	*PF140187*; *PF110161*; *PFB0325c*; *PF110301*; *PF100225*; *PF140341*; *PF140075*; *PF110165*; *PF130141*; *MAL13P1.214*; *PF140346*; *PFE0355c*; *PF140294*; *PF140125*; *PF110162*; *PFB0505c*; *PF140511*; *PF140076*; *PFE0370c*; *PF110147*; *PFB0330c*; *PFF0730c*; *PF140598*; *MAL7P1.27*; *PFI1260c*; *PFB0100c*; *PF080054*; *PF140077*; *MAL13P1.185*; *PF140078*; *PFB0150c*; *PFE1455w*
	c_p1_	Chromosome	10	II; V; VI; VII; VIII; IX; X; XI; XIII; XIV
	c_p2_	Orientation	2	Downstream = −1; Upstream = 1
	c_p3_	Protein function (UniProt)	31	Glutathione s-transferase, putative; Falcipain-2 precursor; Cysteine protease, putative; Spermidine synthase; Orotidine-monophosphate-decarboxylase, putative; Glucose-6-phosphate isomerase; Plasmepsin, putative; Falcipain 2 precursor; l-lactate dehydrogenase; phosphoethanolamine*N*-methyltransferase; cGMP-dependent protein kinase 1, beta isozyme, putative; Serine protease belonging to subtilisin family, putative; Mitogen-activated protein kinase 1; Deoxyhypusine synthase; Falcipain-3; Beta-ketoacyl-acyl carrier protein synthase III precursor, putative; Glucose-6-phosphate dehydrogenase-6-phosphogluconolactonase; Plasmepsin 1 precursor; Subtilisin-like protease precursor, putative; Mitogen-activated protein kinase 2; Enoyl-acyl carrier reductase; Glyceraldehyde-3-phosphate dehydrogenase; Chloroquine resistance transporter, putative; Histone deacetylase; Knob associated histidine-rich protein; Heat shock 70 kDa protein; Plasmepsin 2 precursor; CDK-related protein kinase 6; HAP protein; Protein kinase, putative; Sugar transporter, putative
	c_p4_	ChEMBL target function type	5	Enzyme; Transporter; Epigenetic regulator; Other cytosolic protein; Unclassified Protein

^a^ NL = Number of Levels (unique values) remaining after pre-processing.

**Table 8:** Input variables of the IFPTML models developed.

Variable Type	Symbol	Formula	Categorical Variables	Details
-	*f*(v_ij_)_ref_	n(*f*(v_ij_)_expt_ = 1)/n_j_	c_a0_	Expected value of probability p(*f*(v_ij_) =1)_ref_ for the activity v_ij_ of type c_a0._
MMA_caj_	ΔSh(Drug_i_)_k,caj_	Sh(Drug_i_)_k_– ⟨Sh(Drug)_k,caj_⟩	c_aj_	Variation (Δ) of the information of the structure of the drugin different subsets of multiple categorical variables related to the pharmacological assay c_aj_.
MMA_cdj_	ΔSh(Drug_i_)_k,cdj_	Sh(Drug_i_)_k_ –⟨ Sh(Drug)_k,cdj_⟩	c_dj_	Variation (Δ) of the information of the structure of the drugin different subsets of multiple categorical variables related to the nature and/or accuracy of the data measuredc_dj_.
	ΔSh (Prot_i_)_k,cpj_	Sh(Prot_i_)_k_− ⟨Sh(Prot)_k,cpj_⟩		Variation (Δ) of the information of the sequence of the protein, sequence of the gene, and information about the chromosome for different subsets of multiple categorical variables related tothe nature of the protein target c_pj_.
MMA_cpj_	ΔSh (Gene_i_)_k,cpj_	Sh(Gene_i_)_k_− ⟨Sh(Gene)_k,cpj_⟩	c_pj_	
	ΔSh (Chrom_i_)_k,cpj_	Sh(Chrom_i_)_k_− ⟨Sh(Chrom)_k,cpj_⟩		

## Data Availability

Data supporting the reported results can be found in the Supplementary Materials file.

## Supplementary Materials

The following are available online at https://www.mdpi.com/article/10.3390/ijms222313066/s1.
